# NO-Donation Increases Visceral Circulation in a Porcine Model of Abdominal Hypertension

**DOI:** 10.1007/s12265-022-10299-w

**Published:** 2022-08-29

**Authors:** Per Skoog, Jenny Seilitz, Ioannis Oikonomakis, Tal M. Hörer, Kristofer F. Nilsson

**Affiliations:** 1 Department of Vascular Surgery and Institute of Medicine, Department of Molecular and Clinical Medicine, Sahlgrenska University Hospital and Sahlgrenska Academy, Gothenburg University, Gothenburg, Sweden; 2 Department of Cardiothoracic and Vascular Surgery, Faculty of Medicine and Health, Örebro University, Örebro, Sweden; 3 Department of Surgery, Faculty of Medicine and Health, Örebro University, Örebro, Sweden

**Keywords:** Intraabdominal hypertension, Abdominal compartment syndrome, Intraabdominal pressure, Nitric oxide-donor, Intestinal microcirculation

## Abstract

Intraabdominal hypertension (IAH) is negative for outcome after intensive care. Little research has focused on medical intervention to improve visceral circulation during IAH. A nitric oxide (NO)-donor was compared with placebo in 25 pigs; each pig was randomized into three groups: PDNO (NO-donor), Control (placebo), or Sham. IAH was induced by CO_2_ insufflation to 30 mmHg. Sham group had surgical preparation only. Blood gases, invasive venous and arterial blood pressure, intestinal microcirculation and superior mesenteric blood flow were measured. The PDNO group had significantly increased intestinal microcirculation compared with Controls during IAH (last hour, *P* = 0.009). The mean arterial pressure and abdominal perfusion pressures (APP) were decreased, and the cardiac index were increased in the PDNO group. Also, systemic and pulmonary vascular resistances were lower in the PDNO group compared with Controls. These experimental findings indicate that NO should be further explored with potential application to improve intestinal microcirculation in IAH patients.

**Supplementary Information:**

The online version contains supplementary material available at 10.1007/s12265-022-10299-w.

## Introduction

Intraabdominal hypertension (IAH) and the subsequent condition, abdominal compartment syndrome (ACS) as defined by organ dysfunction, were identified in the 1980s and are often seen after ruptured abdominal aortic aneurysms [1]. Increased awareness of the high prevalence of elevated abdominal pressure and its associated risks has resulted in improved treatment for many intensive care (ICU) patients [2]. Recent studies show that, even without new organ dysfunction, the presence of isolated IAH is strongly linked to survival in ICU patients [3]. The emphasis has been on developing effective treatments for IAH before decompressive laparotomy becomes inevitable [4]. Current strategies aim to reduce intra- and extra-luminal volume in the abdomen and to increase abdominal wall compliance [4, 5]. Increased abdominal pressure can lead to multiorgan failure due to impaired microcirculation in the intestinal wall, with subsequent bacterial translocation and initiation of the inflammatory cascade [6]. Hitherto, little research attention with regard to IAH/ACS has been paid to identifying medical treatments to improve intestinal circulation when the condition can still be reversed and studies of various medical treatment options are warranted.

As a cardiovascular signalling molecule, nitric oxide (NO) has been shown to positively affect both lung and kidney function after aortic cross clamping by having a mitigating effect on the systemic inflammatory response [7, 8]. Organic nitrate NO-donors (e.g., nitroglycerin) have the drawback of tolerance development in both the systemic and the pulmonary circulation [9]. In contrast, organic nitrite NO-donors are associated with less tolerance development and have therefore been presented as an attractive alternative. Previously, we designed a novel NO-donor, comprising the organic mononitrites of 1,2-propanediol, nitrosooxypropanol (PDNO), and found that it was an effective vasodilator in some different models of acute pulmonary hypertension [7, 9–11], and that it ameliorated renal ischemia reperfusion injury [12]. A phase I study in humans is ongoing (EuradaCT No. 2019–001,035-31). The short half-life of PDNO in the circulation makes infusion of PDNO controllable in an intensive care setting.

Little has been explored about the effects of using a vasodilator, e.g., an NO-donor, on organ perfusion and the development of organ dysfunction during elevated abdominal pressure. It may seem paradoxical to use a vasodilator in IAH with the risk of a further reduction in abdominal perfusion pressure. However, it has been shown that nitroglycerin and NO synthase inhibition have beneficial and harmful effects, respectively, on renal perfusion and function in IAH [13, 14].

The aim of the present study was to explore the circulatory and metabolic effects, focusing on the intestines, of a vasodilator in a porcine IAH model. Comparisons were made with the NO-donor PDNO and placebo conditions.

## Methods

### Animals

A randomized experimental study, with an animal model, was performed at the research laboratory in the University Hospital. A total of 25 locally farmed Hampshire and Yorkshire crossbreeds’ domestic pigs were included with the only inclusion criteria: age 3–4 months old and weight 25–36 kg. A block randomization without blinding was used. The report is following the Animal Research: Reporting of In Vivo Experiments (ARRIVE) guidelines. The study was approved by a regional animal ethics committee (ID 124/11, Linköping, Sweden), controlled by a veterinarian, with all experiments to be performed by a certified animal research team in accordance with the guidelines of the European Union Directive 2010/63/EU for the protection of animals used for scientific purposes [15].

### Animal Preparation and Monitoring

The protocol for the anesthesia has been published previously [16]. In brief, the animals were sedated in the morning of each experimental day. Intravenous anesthesia was induced and maintained with continuous propofol (8 mg kg^−1^ h^−1^, Diprivan®, AstraZeneca, Södertälje, Sweden), together with intravenous bolus injections of pethidin (1 mg kg^−1^ h^−1^, Meda, Solna, Sweden), following intubation and volume-controlled ventilation with End tidal CO_2_ monitored. The tidal volumes were set to 10–15 ml kg^−1^ and respiratory frequency to 20–22 min^−1^ at baseline to achieve normoventilation and the tidal volumes were adjusted throughout the experiments to partially compensate for the CO_2_ accumulation in the IAH groups. Airway pressures and tidal volumes were measured with the spirometer unit of the intensive care unit monitor. Muscle relaxants were not given, and control of anesthetic depth was performed throughout the experiments. Ringer’s acetate and 5% glucose solution were infused at 15 ml kg^−1^ h^−1^ and 1.5 ml kg^−1^ h^−1^, respectively, and 750 mg cefuroxime (GSK, Solna, Sweden) was given as a prophylactic antibiotic. At the end of the experiment, the animals were euthanized with a bolus of propofol and pethidine together with the rapid injection of potassium chloride (40 mmol), after which asystolia was confirmed by ECG.

The surgical preparation has been described elsewhere [16]. In brief, both jugular veins were cannulated for fluid and drug administration and pulmonary artery catheterization. The right carotid artery was cannulated for measurement of heart rate and systemic blood pressure, and for blood sampling. A 10-cm midline abdominal incision was made, via which a urinary bladder catheter, a mid-jejunal intraluminal laser Doppler probe, and an ultrasonic transit-time flowmeter around the superior mesenteric artery (SMA) were inserted. The superior mesenteric vein was also catheterized. Via the jugular catheter, a pulmonary arterial catheter was inserted for measurement of pulmonary capillary wedge pressure (PCWP), central venous pressure (CVP), and cardiac output (CO) using the thermodilution technique. Arterial blood samples were analyzed for blood gases, electrolytes, pH, lactate, glucose, and hemoglobin at 37 °C using a GEM Premier 4000 analyzer (Instrumentation Laboratory, Lexington, MA, USA). Transcutaneously, two laparoscopic trocars were inserted into the abdomen for CO_2_ insufflation and visual control of the intraabdominal measurements. The midline incision was closed with an airtight running suture.

### Drugs

The NO-donor PDNO was prepared as has been previously reported [10] and infused using syringe pumps (Alaris CC, Cardinal Health Rulle, Switzerland). A crystalloid hydration solution infused with 1 mL kg^−1^ h^−1^ (Ringer-Acetate, Fresenius Kabi) served as the placebo drug.

### Experimental Protocol

Surgical preparation was followed by a stabilization period of 1 h. The animals were then randomly assigned to 3 groups: (1) the PDNO group (8 animals), (2) the Control group treated with placebo (8 animals), and (3) the Sham group that only had surgical preparation (9 animals). Thereafter, a CO_2_ pneumoperitoneum with intraabdominal pressure of 30 mmHg to induce IAH was established in the PDNO and Control groups. After 2 h of IAH, infusion of PDNO (in a dose of 30 nmol kg^−1^ min^−1^) and the placebo drug was initiated and continued for 4 h until the experiment was ended. The PDNO dose was determined in pilot experiments. The Sham group was prepared identically to the intervention groups, but the animals were not exposed to IAH or drugs. After a stabilization hour (baseline) and at the end of each hour hemodynamic, respiratory and renal variables and intestinal laser Doppler flux were recorded, and blood samples were taken.

### Calculations

The following variables were calculated: body surface area (m^2^) = 0.0734 × weight (kg) 0.656, abdominal perfusion pressure (APP) = systemic mean arterial pressure – intraabdominal pressure, the cardiac index (L min^−1^ m^−2^) = cardiac output · body surface area − 1), the mesenteric flow index (L min^−1^ m^−2^) = mesenteric arterial flow · body surface area − 1), mesenteric oxygen uptake (ml O_2_ m^−2^ min^−1^) = (arterial – mesenteric venous oxygen content blood) · the mesenteric flow index, systemic vascular resistance = (mean systemic arterial pressure – CVP) · the cardiac index − 1), and finally pulmonary vascular resistance = (pulmonary arterial pressure – PCWP) · the cardiac index − 1).

### Statistical Analysis

Data are presented as means and standard errors of the mean (SEM). A linear mixed model for repeated measurements was used for data analysis using an autoregressive correlation structure. Specific measurements were used as outcome variables, and group, time, and their interaction as independent variables. Data showing skewed distributions when evaluated by the Shapiro–Wilks test were log-transformed (laser Doppler flux) before statistical analysis. *P* values of less than 0.05 were regarded as statistically significant for all comparisons. Post hoc test for multiple comparison was performed using Bonferroni correction. All statistical analyses were performed using SPSS version 27 (SPSS Inc., Chicago, IL).

## Results

At baseline all animals displayed normal values, and there were no statistically significant differences between the groups.

### Intraabdominal Pressure and Diuresis

**Fig. 1:**
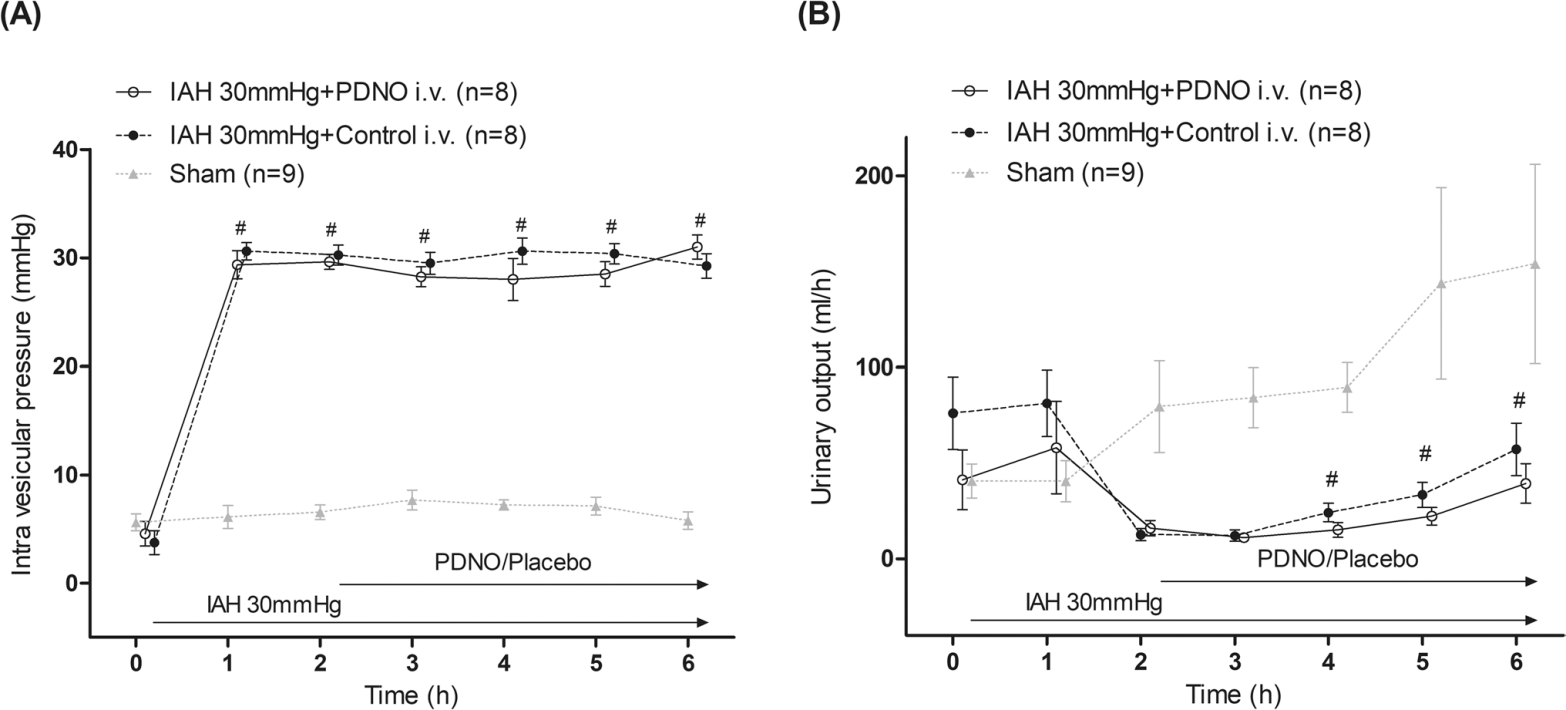
Intravesicular pressure and urinary output. Intravesicular pressure (**A**) and urinary output (**B**) in anesthetized pigs subjected to intraabdominal hypertension by carbon dioxide insufflation. One group of animals had the organic nitrite NO-donor nitrosooxypropanol (PDNO) infused after 2 h (*n* = 8); another group had placebo infused (Control; *n* = 8); and a third group acted as sham with no IAH or drug but surgical preparation in a fashion similar to the intervention groups (Sham, *n* = 9). #Significant difference between an intervention groups and Sham group. *Significant difference between the PDNO and Control groups. Data are means ± standard error of the mean

Intraabdominal pressure measured in the urinary bladder showed the same value as the gas pressure insufflated via the trocars throughout the experiment and served as a control for the model. Urinary output decreased significantly when IAH was induced and stayed at a decreased level throughout the experiment without any difference between the intervention groups (Fig. 1).

### Hemodynamic Parameters and Vascular Resistance

**Fig. 2:**
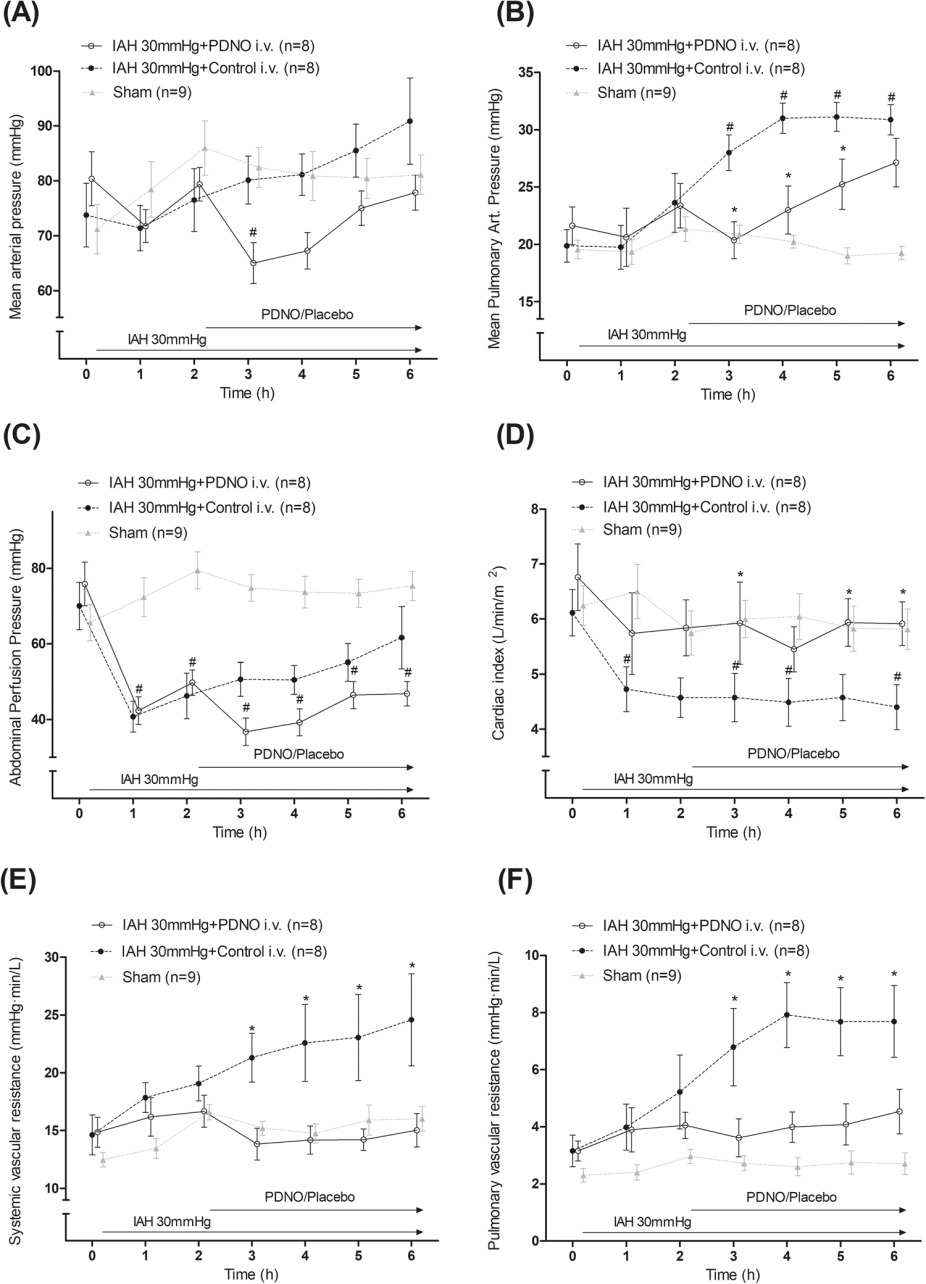
Arterial pressures, abdominal perfusion pressure, cardiac index, and vascular resistance. Mean arterial pressure (MAP; **A**), mean pulmonary arterial pressure (MPAP; **B**), abdominal perfusion pressure (APP; **C**), cardiac index (**D**), systemic vascular resistance (**E**), and pulmonary vascular resistance (**F**) in a model with ventilated and anesthetized pigs. Intraabdominal hypertension was induced by carbon dioxide insufflation. One group of animals had the organic nitrite NO-donor nitrosooxypropanol (PDNO) infused after 2 h (*n* = 8); another group had placebo infused (Control, *n* = 8); and a third group acted as a Sham group with no IAH or drug but surgical preparation in a fashion similar to the intervention groups (Sham, *n* = 9). #Significant difference between an intervention group and Sham. *Significant difference between the PDNO and Control groups. Data are means ± standard error of the mean

Intraabdominal hypertension induced increased heart rate, and there was no difference in heart rate between the intervention groups. IAH decreased cardiac index significantly during the whole experiment in the Control group while PDNO increased cardiac index significantly (Fig. 2). Mean arterial pressure (MAP) was unaffected by IAH initially, but while MAP in the Control group remained unchanged compared with the Sham group throughout the experiment, PDNO decreased MAP significantly during hour 3 compared with the Sham group with restitution the following hours. There was no significant reduction in comparison with the Control group (Fig. 2).

Abdominal perfusion pressure was initially almost halved by IAH compared with the Sham group. APP slowly recovered during the experiment in the Control group while it remained unchanged in PDNO group. There was no significant difference between Control and PDNO during the experiment (Fig. 2).

Systemic vascular resistance was increased by IAH, and the duration of IAH worsened vascular resistance. However, systemic vascular resistance was normalized by PDNO infusion (Fig. 2). Mean pulmonary arterial pressure (MPAP) was also unaffected by IAH initially, but IAH increased MPAP significantly during hours 3–6 in the Control group, and PDNO delayed the MPAP increase. However, in the experiment’s last hour there was no difference between the PDNO and Control groups, and both were significantly increased compared with the Sham group (Fig. 2). Pulmonary vascular resistance was increased by IAH, and throughout the experiment IAH worsened the vascular resistance. Pulmonary vascular resistance was normalized by PDNO infusion (Fig. 2).

**Fig. 3:**
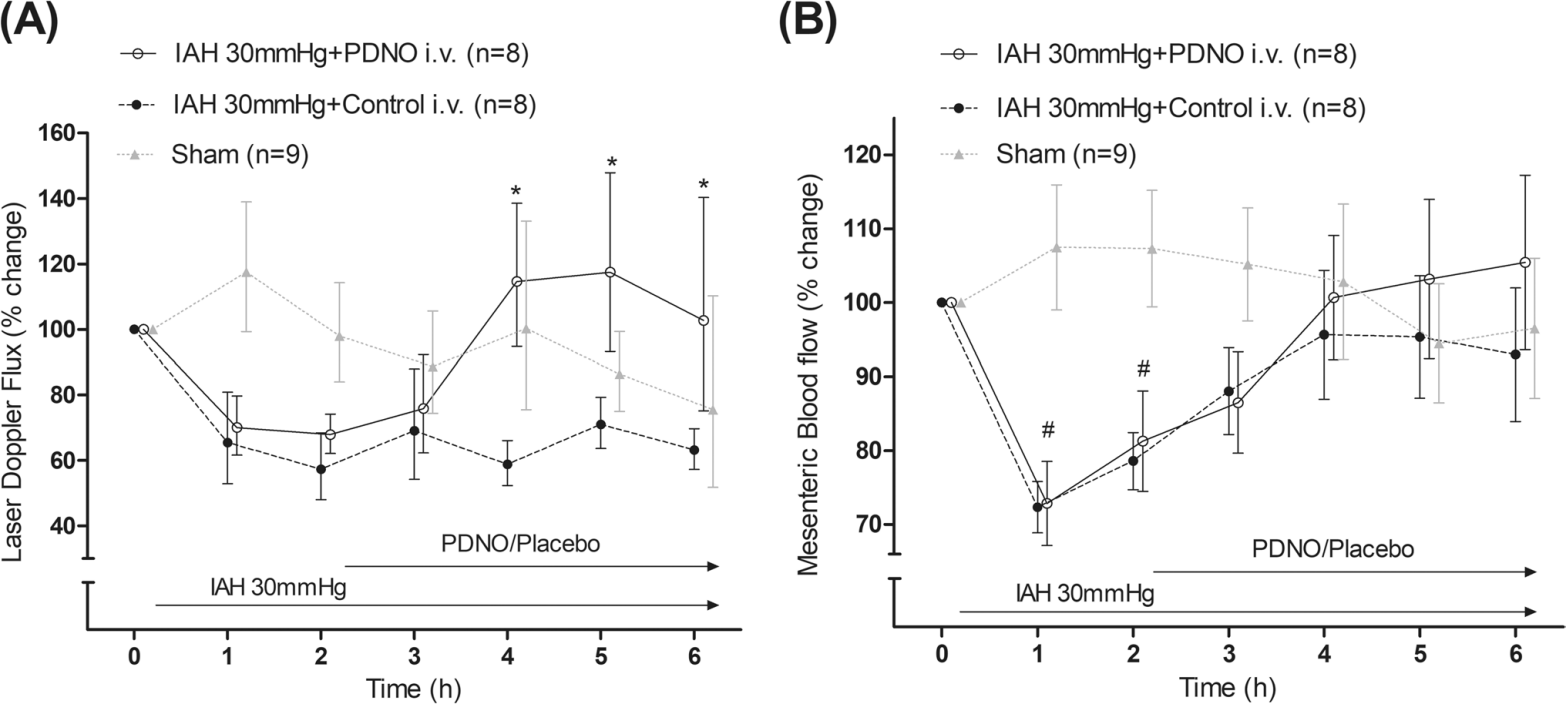
Intestinal microcirculation and superior mesenteric artery flow. Intestinal microcirculation measured with a laser Doppler probe (Laser Doppler Flux; **A**) and superior mesenteric artery flow (**B**) in a porcine model of intraabdominal hypertension (IAH) induced with CO_2_ insufflation during the experiment in anesthetized and ventilated pigs. The organic nitrite NO-donor nitrosooxypropanol (PDNO) was administered in one group of animals (*n* = 8). Placebo was administered in another group (Control, *n* = 8), and 9 animals were a sham group with no IAH or drug infusion but only surgical preparation (Sham, *n* = 9). #Significant difference between an intervention groups and Sham. *Significant difference between the PDNO and Control groups. Data are means ± standard error of the mean

Gut mucosa perfusion was decreased by IAH to approximately 60% of baseline value but was unchanged in the Sham group. PDNO increased the laser Doppler flux to almost 120% during the last 3 h of the experiment, compared with baseline, whereas it was unchanged in the Control group (Fig. 3). Blood flow in the SMA was also decreased significantly by IAH but resituated compared with the Sham group after 4 h. The PDNO group showed significant increase in SMA blood flow between hour 1 and hour 6, which was not seen in the Control group, but there was no statistical difference between groups (Fig. 3).

### Respiratory and Metabolic Parameters

As IAH was induced, peak inspiratory pressure and minute ventilation were increased in the PDNO and Control groups and there was a small, but statistically significant, difference between the PDNO and Control groups at 6 h of IAH (Table 1). Arterial pCO_2_ increased in both the PDNO and Control groups and stayed elevated, with no statistical difference between these groups (Table 1). Arterial pO2 decreased during hours 4 and 6 in PDNO group compared with sham (Table 1). Arterial and mesenteric venous pH and base excess (BE) decreased and arterial lactate concentrations increased with induction of IAH. PDNO did not have any effect on these variables when compared with Control group (Table 1). Mesenteric venous pCO_2_ increased with IAH and was not affected by PDNO infusion.

**Table 1:** Blood gases and metabolic markers in arterial and mesenteric vein blood

	Peak inspiratory pressure (cm H_2_O)	Minute ventilation (L min^−1^)	Arterial pCO_2_ (kPa)	Arterial pO_2_ (kPa)	Arterial pH	Arterial BE (mM)	Arterial lactate (mM)	MV pCO_2_ (kPa)	MV pO_2_ (kPa)	MV pH	MV BE (mM)	MV lactate (mM)
PDNO												
Baseline	19 ± 1	7.5 ± 0.4	5.2 ± 0.2	16.0 ± 1.1	7.45 ± 0.02	3 ± 1	3.4 ± 0.6	6.8 ± 0.4	5.4 ± 0.3	7.35 ± 0.02	2 ± 1	3.4 ± 0.5
2 h	48 ± 3^#^	9.4 ± 0.5	6.9 ± 0.2^#^	14.7 ± 1.3	7.30 ± 0.02^#^	− 1 ± 1^#^	3.9 ± 1.0^#^	10.6 ± 0.6^#^	5.0 ± 0.2	7.17 ± 0.03^#^	− 1 ± 1^#^	3.7 ± 0.9^#^
4 h + PDNO	49 ± 3^#^	11.0 ± 0.8^#^	7.0 ± 0.4^#^	12.2 ± 1.0^#^	7.31 ± 0.02^#^	0 ± 1^#^	3.5 ± 0.8^#^	9.9 ± 0.5^#^	5.3 ± 0.3	7.21 ± 0.02^#^	1 ± 1^#^	3.2 ± 0.8^#^
6 h + PDNO	53 ± 3^*#^	11.5 ± 0.9^#^	7.2 ± 0.3^#^	11.0 ± 0.8^#^	7.34 ± 0.01^#^	3 ± 1^#^	2.2 ± 0.5	10.1 ± 0.4^*#^	5.5 ± 0.2	7.23 ± 0.02^#^	1 ± 1^#^	2.3 ± 0.5
Control												
Baseline	20 ± 1	7.7 ± 0.5	5.4 ± 0.3	15.0 ± 1.0	7.46 ± 0.02	5 ± 1	3.2 ± 0.4	6.8 ± 0.3	5.0 ± 0.5	7.38 ± 0.01	4 ± 0	3.2 ± 0.4
2 h	51 ± 2^#^	9.7 ± 0.6	6.8 ± 0.5^#^	15.5 ± 1.0	7.31 ± 0.02^#^	− 1 ± 1^#^	3.6 ± 0.9^#^	12.1 ± 1.0^#^	5. 2 ± 0.5	7.15 ± 0.03^#^	0 ± 2^#^	4.0 ± 1.0^#^
4 h + Placebo	52 ± 2^#^	10.4 ± 0.5^#^	7.2 ± 0.5^#^	13.1 ± 0.5	7.33 ± 0.02^#^	2 ± 1^#^	2.3 ± 0.5	11.3 ± 0.6^#^	5.9 ± 0.6	7.18 ± 0.01^#^	2 ± 1	2.6 ± 0.5
6 h + Placebo	57 ± 1^#^	11.2 ± 0.6^#^	6.9 ± 0.5^#^	13.8 ± 1.1	7.34 ± 0.02^#^	2 ± 1^#^	1.9 ± 0.7	11.6 ± 0.8^#^	6.2 ± 0.5	7.18 ± 0.03^#^	3 ± 1^#^	2.3 ± 0.7
Sham												
Baseline	19 ± 1	7.9 ± 0.4	5.2 ± 0.2	16.4 ± 1.1	7.45 ± 0.02	3 ± 1	2.1 ± 0.3	7.9 ± 0.6	6.2 ± 0.6	7.30 ± 0.04	2 ± 1	2.7 ± 0.5
2 h	20 ± 1	8.2 ± 0.4	4.9 ± 0.1	15.9 ± 0.9	7.49 ± 0.01	4 ± 1	1.0 ± 0.1	6.8 ± 0.2	5.5 ± 0.3	7.38 ± 0.02	4 ± 1	1.3 ± 0.2
4 h	20 ± 1	8.1 ± 0.4	4.7 ± 0.1	15.7 ± 0.8	7.52 ± 0.01	6 ± 1	0.8 ± 0.1	6.7 ± 0.2	5.2 ± 0.3	7.40 ± 0.02	5 ± 1	1.2 ± 0.2
6 h	20 ± 1	7.8 ± 0.3	4.8 ± 0.1	14.6 ± 1.0	7.54 ± 0.01	8 ± 1	0.7 ± 0.1	7.1 ± 0.5	5.0 ± 0.3	7.40 ± 0.03	7 ± 1	1.4 ± 0.2

Blood gases, pH, and BE in systemic arterial blood and mesenteric vein blood (MV) in a model of intraabdominal hypertension (IAH) induced with CO_2_ insufflation during the experiment in anesthetized and ventilated pigs. Organic nitrite NO-donor nitrosooxypropanol (PDNO) (*n* = 8) and Controls (*n* = 8) compared with Sham without IAH (*n* = 9)

^#^Statistical difference when comparing a variable with sham

^*^Statistical difference when comparing PDNO with Controls. Data are means ± standard error of the mean

## Discussion

This study, with a porcine model of IAH, demonstrated that the NO-donor PDNO not only counteracted the negative effect of increased abdominal pressure on the intestinal microcirculation measured by laser Doppler but also increased microcirculation compared with baseline. The positive effect on intestinal microcirculation was seen at the same time as abdominal perfusion pressure decreased due to falling MAP during administration of the NO-donor. The NO-donor PDNO also increased cardiac index. Also, systemic and pulmonary vascular resistances were lowered by the infusion of a NO-donor. During the short experiment, no effect on diuresis was seen; nor was there any significant change in the metabolic markers from the visceral organ (see Supplementary Table).

The cardiovascular effects of IAH are complex, and our knowledge is not comprehensive. It is known that IAH has an impact on circulation, by changing preload, heart contractility, and afterload [17]. Increased IAH continued to the thoracic compartment is also known to compress the heart, which results in decreased end-diastolic volumes. All this, alongside compressed vascular beds with subsequent increased systemic afterload and activation of the renin–angiotensin–aldosterone pathway, results in decreased cardiac index [18].

Mean arterial pressure is known to be initially unaffected when abdominal pressure rises, due to shunting past the abdomen, but usually drops with sustained IAH. In this study, PDNO significantly decreased MAP initially, but it was restored after 4 h. The effect on systemic vascular resistance was significant with PDNO infusion. Compared with the sham group, IAH prompted a sharp increase in vascular resistance on the arterial side. However, PDNO completely counteracted the increase in systemic vascular resistance, an effect that was not present in the Control group. The known IAH effect on MPAP was confirmed in the present study [7]. It was evident that PDNO significantly delayed the MPAP increase whereas placebo did not. Pulmonary vascular resistance showed changes similar to systemic vascular resistance: a marked increase with the induction of IAH, and a significant PDNO effect that erased the difference between the Sham group and the PDNO group.

Our finding in the current study was that PDNO maintained an almost normal cardiac index, most likely due to reduced afterload, by perhaps counteracting systemic vasoconstriction provoked by sympathetic stimulation and the renin–angiotensin–aldosterone axis [17, 18].

The effect of normalized afterload in IAH and ACS is interesting regarding the intention to make an early intervention to avoid the development of ACS. If reduced vascular resistance leads to less shunting of blood past the intestines, the perfusion of mucosa can be maintained, and the risk of bacterial translocation is reduced, the risk of local intestinal ischemia may decrease and a vicious circle may be broken.

Abdominal perfusion pressure (APP) dropped drastically when IAH was initiated, and PDNO prohibited the restitution seen in Controls compared with sham. However, APP did not differ significantly between Control and PDNO during the experiment. In spite of the significant drop in APP induced by PDNO, no negative metabolic changes were seen, although this experiment was short. Perfusion pressure is indeed important but has to be evaluated together with peripheral vascular resistance and flow in order to obtain the overall picture of the end organ physiology during IAH.

Microcirculation in the intestine and perfusion of the intestinal mucosa were investigated in the present experiment by means of laser Doppler. Laser Doppler has previously been used to determine gut microcirculation and has been shown to be in good agreement with other methods of measuring intestinal perfusion, such as microdialysis [6]. In the present study, IAH significantly reduced bowel perfusion. This is consistent with previous research and has been explained by the combination of decreased CO, increased vascular resistance and increased venous stasis with decreased venous drainage from the intestine, as seen with IAH [19]. When infusion of PDNO started, gut microcirculation recovered and the laser Doppler value increased beyond baseline, whereas the Control group retained reduced microcirculation in the mucosa compared with controls during the remainder of the experiment. This finding is of special interest as a simultaneous APP reduction was noted. The increased intestinal perfusion, we believe, was also marked by an increase in blood flow in the superior mesenteric artery during the course of the experiment in the PDNO group. However, no intergroup difference was seen between the PDNO and the Control groups regarding mesenteric blood flow. It is interesting to find that infusion of a vasodilator drug can increase intestinal perfusion in an IAH model. It is tempting to envisage areas of use in the early treatment of high abdominal pressure in order to reverse unfavorable processes in critically ill patients. In cardiac research it is furthermore known that NO is cardioprotective by decreasing the negative effect of angiotensin II [20]. With future studies including end-organ monitoring, the NO effect on the visceral organs must be further explored. PDNO’s positive effect on intestinal microcirculation came with a time lag of roughly 3 h. The half-life and thereby time to steady state of the NO donor is very short and cannot by itself explain this lag in effect. After IAH induction and without any therapy, compensatory circulatory mechanisms take place where shunting is probably one of the most significant. The lag in PDNO effect may be interconnected to the effectiveness of these natural compensatory mechanisms. If it is the case that NO leads to better perfusion of the intestines without the shunt mechanisms being initiated, is unclear and remains to be investigated in future research.

One of the main limitations of this study lies in the short time span of the intervention, which may have affected our ability to explore the metabolic consequences of the NO-donor. Also, the fact that the model directly induced IAH with 30 mmHg means that most cardiovascular changes became extreme, which does not reflect a clinical scenario where it might be relevant to administer a NO-donor. A histopathological analysis of the intestinal wall could have strengthened the microcirculatory finding. Moreover, only the initial phase of the physiological changes after induction of IAH was explored in this experiment and in future research it is of interest to investigate the responses to reperfusion after normalization of abdominal pressure.

In conclusion, we found that the NO-donor PDNO decreased systemic and pulmonary vascular resistances, elevated the cardiac index score and, most importantly, counteracted decreased microcirculatory blood flow in the intestinal mucosa during IAH in a porcine model. These findings warrant further research to explore a potential application to improve intestinal microcirculatory perfusion in intensive care patients with intraabdominal hypertension.

## Clinical Relevance

As the presence of intraabdominal hypertension is negatively linked to the survival of ICU patients and there has been little efforts to find noninvasive therapies to reverse the course of the disease early, this exploratory experimental work has clinical relevance. Based on the results of this study, it is not possible to justify clinical use of NO treatment during IAH. However, the results show the possibility that NO can facilitate intestinal perfusion during IAH and open up for further research on this treatment option.

## Supplementary Information

Supplementary Table (DOCX 33.4 KB)

## Abbreviations

ACS: Abdominal compartment syndrome
APP: Abdominal perfusion pressure
BE: Base excess
CO: Cardiac output
CO2: Carbon dioxide
CVP: Central venous pressure
ECG: Electrocardiogram
IAH: Intraabdominal hypertension
ICU: Intensive care unit
ITP: Intrathoracic pressure
kPa: Kilopascal
MAP: Mean arterial pressure
mM: Millimolar
MPAP: Mean pulmonary arterial pressure
NO: Nitric oxide
O2: Oxygen
PCWP: Pulmonary capillary wedge pressure
PDNO: Nitrosooxypropanol
SEM: Standard error of the mean
SMA: Superior mesenteric artery
