# Supplementary material for: NO-Donation Increases Visceral Circulation in a Porcine Model of Abdominal Hypertension
Source: J Cardiovasc Transl Res. 2022 Aug 29;16(1):42–50. doi: 10.1007/s12265-022-10299-w (PMC9944725; doi:10.1007/s12265-022-10299-w)
Supplement: Supplementary file 1 — (DOCX 33.4 KB) [file 12265_2022_10299_MOESM1_ESM.docx]

P-values for vascular resistance, hemodynamic- and metabolic parameters

|  | Baseline | Hour 1 | Hour 2 | Hour 3 | Hour 4 | Hour 5 | Hour 6 |
| --- | --- | --- | --- | --- | --- | --- | --- |
| Intra vesicular pressure (mmHg) |  |  |  |  |  |  |  |
| PDNO vs. Control | 1 | 1 | 1 | 0.15 | 0.33 | 0.627 | 0.054 |
| Control vs. Sham | 1 | <0.001 | <0.001 | 0.003 | 0.003 | 0.027 | 0.038 |
| PDNO vs. Sham | 0.408 | <0.001 | <0.001 | <0.001 | <0.001 | <0.001 | <0.001 |
| Urinary output (ml/h) |  |  |  |  |  |  |  |
| PDNO vs. Control | 0.795 | 1 | 1 | 1 | 1 | 1 | 1 |
| Control vs. Sham | 0.735 | 0.552 | 0.09 | 0.06 | 0.102 | <0.001 | 0.006 |
| PDNO vs. Sham | 1 | 1 | 0.117 | 0.054 | 0.048 | <0.001 | <0.001 |
| Mean Arterial Pressure (mmHg) |  |  |  |  |  |  |  |
| PDNO vs. Control | 0.975 | 1 | 1 | 0.081 | 0.126 | 0.363 | 0.06 |
| Control vs. Sham | 1 | 0.84 | 0.444 | 1 | 1 | 1 | 0.411 |
| PDNO vs. Sham | 0.489 | 0.918 | 0.933 | 0.027 | 0.117 | 1 | 1 |
| Mean Pulmonary Arterial Pressure (mmHg) |  |  |  |  |  |  |  |
| PDNO vs. Control | 1 | 1 | 1 | 0.003 | <0.001 | 0.024 | 0.339 |
| Control vs. Sham | 1 | 1 | 0.855 | 0.003 | <0.001 | <0.001 | <0.001 |
| PDNO vs. Sham | 1 | 1 | 1 | 1 | 0.585 | 0.009 | <0.001 |
| Abdominal Perfusion Pressure (mmHg) |  |  |  |  |  |  |  |
| PDNO vs. Control | 1 | 1 | 1 | 0.15 | 0.33 | 0.657 | 0.054 |
| Control vs. Sham | 1 | <0.001 | <0.001 | 0.003 | 0.003 | 0.027 | 0.138 |
| PDNO vs. Sham | 0.408 | <0.001 | <0.001 | <0.001 | <0.001 | <0.001 | <0.001 |
| Cardiac Index (L/min/m^2^) |  |  |  |  |  |  |  |
| PDNO vs. Control | 0.312 | 0.193 | 0.068 | 0.045 | 0.135 | 0.04 | 0.048 |
| Control vs. Sham | 0.852 | 0.013 | 0.078 | 0.028 | 0.015 | 0.076 | 0.046 |
| PDNO vs. Sham | 0.411 | 0.235 | 0.906 | 0.892 | 0.353 | 0.725 | 0.974 |
| Systemic Vascular Resistance (mmHg·min/L) |  |  |  |  |  |  |  |
| PDNO vs. Control | 1 | 1 | 0.762 | 0.009 | 0.003 | 0.003 | <0.001 |
| Control vs. Sham | 1 | 0.462 | 1 | 0.09 | 0.015 | 0.024 | 0.006 |
| PDNO vs. Sham | 1 | 1 | 1 | 1 | 1 | 1 | 1 |
| Pulmonary Vascular Resistance (mmHg·min/L) |  |  |  |  |  |  |  |
| PDNO vs. Control | 1 | 1 | 0.717 | 0.006 | <0.001 | 0.003 | 0.009 |
| Control vs. Sham | 1 | 0.711 | 0.108 | <0.001 | <0.001 | <0.001 | <0.001 |
| PDNO vs. Sham | 1 | 0.444 | 1 | 1 | 0.693 | 0.702 | 0.246 |
| Laser Doppler Flux (% change) |  |  |  |  |  |  |  |
| PDNO vs. Control | 1 | 1 | 1 | 1 | 0.009 | 0.018 | 0.035 |
| Control vs. Sham | 1 | 0.093 | 0.291 | 1 | 0.06 | 1 | 0.483 |
| PDNO vs. Sham | 1 | 0.216 | 1 | 1 | 1 | 0.192 | 0.27 |
| Mesenteric Blood Flow (% change) |  |  |  |  |  |  |  |
| PDNO vs. Control | 1 | 1 | 1 | 1 | 0.883 | 0.473 | 0.27 |
| Control vs. Sham | 1 | 0.003 | 0.024 | 0.324 | 0.693 | 1 | 1 |
| PDNO vs. Sham | 1 | 0.006 | 0.048 | 0.24 | 1 | 0.489 | 0,57 |
| Arterial pCO2 (kPa) |  |  |  |  |  |  |  |
| PDNO vs. Control | 1 |  | 1 |  | 1 |  | 1 |
| Control vs. Sham | 1 |  | <0.001 |  | <0.001 |  | <0.001 |
| PDNO vs. Sham | 1 |  | <0.001 |  | <0.001 |  | <0.001 |
|  |  |  |  |  |  |  |  |
| Arterial pO_2_ (kPa) |  |  |  |  |  |  |  |
| PDNO vs. Control | 1 |  | 1 |  | 1 |  | 0.171 |
| Control vs. Sham | 0.942 |  | 1 |  | 0.174 |  | 1 |
| PDNO vs. Sham | 1 |  | 1 |  | 0.042 |  | 0.042 |
| Arterial pH |  |  |  |  |  |  |  |
| PDNO vs. Control | 1 |  | 1 |  | 1 |  | 1 |
| Control vs. Sham | 1 |  | <0.001 |  | <0.001 |  | <0.001 |
| PDNO vs. Sham | 1 |  | <0.001 |  | <0.001 |  | <0.001 |
| Arterial BE (mM) |  |  |  |  |  |  |  |
| PDNO vs. Control | 0.951 |  | 1 |  | 1 |  | 1 |
| Control vs. Sham | 0.888 |  | 0.003 |  | 0.009 |  | <0.001 |
| PDNO vs. Sham | 1 |  | <0.001 |  | <0.001 |  | <0.001 |
| Arterial lactate (mM) |  |  |  |  |  |  |  |
| PDNO vs. Control | 1 |  | 1 |  | 0.435 |  | 1 |
| Control vs. Sham | 0.489 |  | 0.003 |  | 0.162 |  | 0.369 |
| PDNO vs. Sham | 0.249 |  | <0.001 |  | 0.003 |  | 0.18 |
| MV pCO_2_ (kPa) |  |  |  |  |  |  |  |
| PDNO vs. Control | 1 |  | 0.156 |  | 0.171 |  | 0.048 |
| Control vs. Sham | 0.369 |  | <0.001 |  | <0.001 |  | <0.001 |
| PDNO vs. Sham | 0.381 |  | <0.001 |  | <0.001 |  | 0.003 |
| MV pO_2_ (kPa) |  |  |  |  |  |  |  |
| PDNO vs. Control | 1 |  | 1 |  | 0.861 |  | 0.756 |
| Control vs. Sham | 0.183 |  | 1 |  | 0.645 |  | 0.186 |
| PDNO vs. Sham | 0.489 |  | 0.981 |  | 1 |  | 1 |
| MV pH |  |  |  |  |  |  |  |
| PDNO vs. Control | 1 |  | 1 |  | 1 |  | 0.165 |
| Control vs. Sham | 0.057 |  | <0.001 |  | <0.001 |  | <0.001 |
| PDNO vs. Sham | 0.318 |  | <0.001 |  | <0.001 |  | <0.001 |
| MV BE (mM) |  |  |  |  |  |  |  |
| PDNO vs. Control | 0.762 |  | 1 |  | 1 |  | 1 |
| Control vs. Sham | 0.36 |  | 0.045 |  | 0.069 |  | 0.048 |
| PDNO vs. Sham | 1 |  | 0.006 |  | 0.009 |  | 0.015 |
| MV lactate (mM) |  |  |  |  |  |  |  |
| PDNO vs. Control | 1 |  | 1 |  | 1 |  | 1 |
| Control vs. Sham | 1 |  | 0.003 |  | 0.225 |  | 0.645 |
| PDNO vs. Sham | 1 |  | 0.009 |  | 0.036 |  | 0.96 |
